# “Thinking About Thinking” in Insomnia Disorder: The Effect of Cognitive-Behavioral Therapy for Insomnia on Sleep-Related Metacognition

**DOI:** 10.3389/fpsyg.2021.705112

**Published:** 2021-09-09

**Authors:** Andrea Galbiati, Marco Sforza, Alessandro Scarpellino, Andrea Salibba, Caterina Leitner, Giada D’Este, Samantha Mombelli, Luigi Ferini-Strambi, Vincenza Castronovo

**Affiliations:** ^1^Vita-Salute San Raffaele University, Milan, Italy; ^2^Department of Clinical Neurosciences, Neurology – Sleep Disorders Center, IRCCS San Raffaele Scientific Institute, Milan, Italy

**Keywords:** insomnia, cognitive-behavioral therapy for insomnia, metacognition, dysfunctional beliefs, worry

## Abstract

Metacognition is defined as the ability to reflect on one’s mental state and to govern thoughts and beliefs. Metacognitive dysfunctions are typical of several psychopathologic conditions, and also a feature of insomnia disorder, possibly playing a crucial role in its genesis and maintenance. In the context of insomnia, metacognition describes how individuals react to their own sleep-related thoughts and beliefs, boosting the hyperarousal state experienced by these patients. Up to now, no studies evaluated the effect of cognitive behavioral therapy for insomnia (CBT-I) on metacognitive functioning. Therefore, the aim of our study was to evaluate the effect of CBT-I administered in group format in patients with insomnia disorder. As expected, all patients showed significant improvements in both insomnia and sleep diary parameters after treatment. Furthermore, an improvement was observed also in dysfunctional metacognitive levels, assessed by means of the Metacognitions Questionnaire-Insomnia (MCQ-I). However, 63% of patients still showed a MCQ-I score above the clinical cutoff after treatment. Dividing the sample on the basis of MCQ-I questionnaire scores after CBT-I, we found that patients, who still presented metacognitive impairment, received significant beneficial effects from CBT-I both on insomnia symptoms and on dysfunctional beliefs, but not on dysfunctional metacognitive functioning. These findings suggest that metacognition should be carefully evaluated in insomnia patients and further studies are needed to evaluate long-term implications of this remaining dysfunction.

## Introduction

Insomnia disorder is a highly prevalent disorder, as it is the most common sleep disorder encountered in clinical practice and the second most prevalent mental disorder in the European countries (Wittchen et al., 2011; Sateia, 2014). It is characterized by difficulties in initiating and maintaining sleep and early morning awakenings associated with a complaint of sleep dissatisfaction and important daytime consequences that interfere with working and social functioning (American Psychiatric Association, 2013; Sateia, 2014). Moreover, insomnia represents a risk factor for the development of mental disorders (depression, anxiety, alcohol abuse and psychosis; Hertenstein et al., 2019) as well as medical condition (Vgontzas et al., 2013). Importantly, although naively considered a nighttime disorder, insomnia is now universally deemed a daytime 24-h disorder (Ferini-Strambi et al., 2021). Indeed, one of the most accredited hypothesis for the genesis and the maintenance of the disorder is the hyperarousal model. This model described insomnia patients as characterized by increased neurophysiological and cognitive activities that impede relaxation and physiological drive to sleep resulting in prolonged sleep latencies and increased nocturnal awakenings (Riemann et al., 2010).

In this context, a refinement of different models regarding cognitive activity in insomnia is of utmost importance for developing new etiopathogenetic hypotheses but also for fine tuning therapy. Accordingly, a metacognitive model for insomnia has been proposed (Ong et al., 2012). Metacognition can be defined as a form of awareness or knowledge regarding one’s own mental state and cognitive processes, such as the ability of governing or regulating thoughts and beliefs (Flavell, 1976). The striking relevance of metacognitive theory is to shift the focus from thoughts’ contents to the processes that govern such cognition, such as repetitiveness and intrusiveness, in order to manage the unhelpful thinking styles. In other words, we could define metacognitive level as a form of “thoughts on thoughts,” or beliefs regarding the usefulness of specific cognitive processes (Wells, 2009b). In the field of insomnia, Ong et al. (2012) differentiates between cognitions and metacognitions, defined two specific levels of cognitive arousal. “Primary arousal” refers to cognitive activity and contents directly interfering with sleep. This category comprises thoughts and beliefs affecting sleep or related to daytime consequence of disrupted sleep. “Secondary arousal” concerns how individuals react to sleep-related thoughts and beliefs. This category includes the interpretative value related to cognitive activity, such as emotional response associated with thoughts, the levels of attachment to these and their meaning. This secondary component magnifies the negative emotional evaluation biasing the attentional system of the subject toward sleep-related cognition generated at the former level. As an example, the thought/belief “I need 8h of sleep to function well the next day” might generate a form of primary arousal interfering with sleep drive when the subject is trying to sleep. In addition, cognitive rumination and worry caused by the attentional bias to this belief amplify negative emotion, boosting secondary arousal, and impeding the automatic regulation of sleep–wake patterns typical of good sleepers (Espie et al., 2006). According to this view, “secondary arousal” represents the core feature for the maintenance of insomnia, impeding subjects to create alternative coping strategies.

Previous research on this topic showed that metacognition represents a core feature of insomnia disorder confirming that dysfunctional metacognitive functioning is strictly associated to poor sleep quality in this disorder when compared to other sleep disorders such as obstructive sleep apnea (Palagini et al., 2014). Specifically, metacognitive sleep-related beliefs are strictly related to stress-related sleep reactivity, i.e., the vulnerability to sleep disturbance when exposed to stress (Palagini et al., 2016a). Remarkably, sleep-related metacognitive processes have stronger effect in modulating hyperarousal in comparison to sleep reactivity (Palagini et al., 2016b). Moreover, although both sleep-related cognitive and metacognitive processes are related to somatic and cognitive pre-sleep arousal, when considering insomnia disorder’s duration, the association between metacognitive processes and pre-sleep arousal is more prominent, thus highlighting their role in the chronicization of the disease (Palagini et al., 2017). Interestingly, an interaction between metacognitive beliefs related to sleep difficulties and subjective sleep quality in older subjects has been described (Sella et al., 2019).

Taken together, these findings underscore the crucial role played by metacognition in the genesis and maintenance of insomnia disorder. However, up to now, no studies investigated the effect of cognitive-behavioral therapy for insomnia (CBT-I) on dysfunctional metacognition. Therefore, the aim of our study was to (i) confirm the presence of metacognitive impairment in patients with insomnia compared to healthy subjects and (ii) to evaluate the effect of a group CBT-I in patients with insomnia disorder on sleep-related metacognition. By means of two specific validated scale, Dysfunctional Beliefs and Attitudes about Sleep (DBAS; Morin et al., 2007) and Metacognitions Questionnaire – Insomnia (MCQ-I; Waine et al., 2009), we aimed to assess the effect of CBT-I both on -primary (DBAS) and -secondary (MCQ-I) arousal postulated in the metacognitive model of insomnia (Ong et al., 2012). Our hypothesis is that despite a robust effect in ameliorating sleep and sleep-related cognition, CBT-I might have a reduced effect on dysfunctional metacognition due to a lack of a specific treatment component incorporated in CBT-I protocol. CBT-I does not include a protocol aimed to treat metacognitive dysfunction; on the contrary it includes a session of cognitive therapy aimed to challenge and restructure dysfunctional beliefs and cognition regarding sleep. Therefore, treating only sleep-related dysfunctional beliefs and not targeting metacognitive functioning might not be sufficient. This could represent a limitation, not directly for therapy outcome, but for the occurrence of relapses in the long term. Indeed, the absence of a specific metacognitive treatment may not limit the effect of CBT-I on patients’ sleep, that is strongly ascribable to treatment’s behavioral component (Maurer et al., 2020), but may favor the re-emergence of uncontrollable maladaptive strategies (e.g., pre-sleep worry and rumination) thus leading to relapses.

## Materials and Methods

### Participants

Between 2019 and 2020, 27 consecutive insomnia patients (52% females, mean age 46.37±13.67years, age range 18–71years, disease duration of 10.04±8.79years) and 23 healthy controls (63% females, mean age 33.00±13.77years) were enrolled in this study. Patients visiting the Sleep Disorders Center of San Raffaele Hospital, Milan, were recruited for the purposes of this study, after a clinical evaluation performed by physician experts in sleep medicine who diagnosed insomnia disorder according to diagnostic criteria of ICSD 3 (Sateia, 2014). Patients taking medications and/or with major psychiatric comorbidities and/or with untreated medical conditions were excluded from the study. Healthy subjects were recruited with the same exclusion criteria and on the basis of an Insomnia Severity Index (ISI) score <10 (Morin et al., 2011). All participants provided written informed consent to the experimental procedure, which was previously approved by the local ethical committee (protocol number of ethics approval: 188/INT/2020).

### Procedures

CBT-I consisted of a multicomponent treatment of seven 90-min group sessions (Spielman et al., 1987; Lichstein, 1988; Espie, 1991; Hauri, 1991; Morin, 1993). A licensed psychotherapist certified in sleep medicine conducted group sessions. The sessions were held weekly or biweekly depending on session content. The first and second sessions concerned introduction and education on the principles of regulation of sleep–wake system. In particular, the aim of the first session was to provide a general overview and define treatment goals, based on each patient’s insomnia characteristics. The second session consisted of psychoeducation on sleep hygiene with the aim of emphasizing the importance of sleep and developing awareness about the factors that maintain the disorder. In the third session, strategies for reducing cognitive and emotional arousal were provided through deep-breathing relaxation exercise and cognitive strategies to reduce excessive thinking in bed. In the fourth session, stimulus control and sleep restriction techniques were introduced followed by an individual treatment plan that each subject received from the therapist. Session five dealt with how to keep improving sleep efficiency. Session six concerned cognitive restructuring of dysfunctional beliefs about sleep and the last session concerned review and relapse prevention.

All patients completed a comprehensive questionnaires battery before and after CBT-I aimed to evaluate insomnia severity, chronotype, daytime sleepiness, sleep-related metacognitions, sleep-related dysfunctional beliefs, stress, global health, anxiety, and depression symptoms.

- ISI (Bastien, 2001; Morin et al., 2011; Castronovo et al., 2016): ISI is a self-report questionnaire that assesses impact and severity of insomnia. It consists of seven items evaluating: (1) the severity of sleep onset, sleep maintenance and early morning awakenings; (2) satisfaction level with current sleep pattern; (3) interference of sleep difficulties with daytime functioning; (6) noticeability of sleep problems by others; (7) distress caused by the sleep difficulties. Each item is rated on a five-point Likert scale (0–4) with a total score ranging from 0 to 28. Higher scores indicate greater insomnia severity. *α*=0.75.
- Morningness–Eveningness Questionnaire (MEQ; Horne and Ostberg, 1976; Natale et al., 2006): MEQ is a 19-item self-administered questionnaire which assesses morning- (59–86), intermediate- (42–58) and evening-chronotype (16–41) subjects. The total score ranges from 16 to 86. *α*=0.68.
- Epworth Sleepiness Scale (ESS; Johns, 1991; Vignatelli et al., 2003): ESS is a eight-item self-administered questionnaire that evaluates the subject’s sleep propensity during everyday life, thus providing an index of daytime sleepiness. The total score ranges from 0 to 24, with higher scores indicating greater levels of daytime sleepiness. *α*=0.88.
- Metacognitions Questionnaire-Insomnia (MCQ-I; Waine et al., 2009; Sella et al., 2016): MCQ-I is self-report questionnaire that consists of 60 items evaluating metacognitive beliefs about sleep in insomnia patients. Each item is rated on a four-point Likert scale (from 0: “do not agree” to 4: “agree very much”). Waine et al. (2009) reported that a cut-off of 110 correctly differentiated insomnia patients from normal sleepers and the discriminant validity, scale sensitivity and specificity of the questionnaire have been demonstrated. *α*=0.95.
- Dysfunctional Beliefs and Attitudes about Sleep Scale (DBAS-16; Coradeschi et al., 2000; Morin et al., 2007): DBAS-16 is a self-report scale which consists of 16 items assessing cognitions about sleep and it is composed of four factors: (1) consequences of insomnia; (2) worry about insomnia; (3) sleep expectations; and (4) beliefs about medication. Each item is rated on a visual analogue scale from 0 (strongly disagree) to 10 (strongly agree). The total score ranges from 0 to 160, with higher scores indicating greater presence of dysfunctional beliefs about sleep. *α*=0.77.
- Perceived Stress Scale (PSS; Cohen et al., 1983; Cohen, 1988; Mondo et al., 2019): PSS is a 10-item scale which assesses perceived stress during the past month. Each item is rated on a five-point Likert scale form 0 (never) to 4 (very often). The total score ranges from 0 to 40, with higher scores indicating greater perceived stress. *α*=0.83.
- General Health Questionnaire-12 (GHQ-12; Goldberg and Hillier, 1979; Goldberg, 1988; Giorgi et al., 2014): GHQ-12 is a 12-item self-administered questionnaire for the assessment of mental health and the detection of psychological distress. Each item is scored on a four-point Likert Scale (0–3). The total score ranges from 0 to 36, with higher scores indicating greater psychological distress. *α*=0.85
- Beck Depression Inventory (BDI-II): BDI-II is a self-administered questionnaire, composed of 21 items on a four-point Likert scale (Beck et al., 1996). This questionnaire is design for diagnosis of depression and to assess depression symptom severity. The scores are divided into two subscales: affective symptoms (eight items) and somatic symptoms (13 items). BDI-II scores range between 0 and 63, with “minimal range” of 0–13, “mild” 14–19, “moderate” 20–28, and “severe” 29–63. *α*=0.89.
- State–Trait Anxiety Inventory (STAI): STAY provides a measure of trait- and state- anxiety by means of two scales that can be used independently (Spielberger et al., 1970). The scale STAI-Y-1 evaluates how the subject feels “at this moment” while STAI-Y-2 assesses patient’s trait. Both scales consist of 20 items with a total score that ranges from 20 to 80. Of note, higher values indicate higher level of anxiety. *α*=from 0.86 to 0.95.

A sleep diary was compiled by patients from the week before the first CBT-I session and throughout the whole period of the treatment following the guidelines provided by Carney et al. (2012) for the consensus sleep diary (Carney et al., 2012). Sleep parameters extracted from sleep diaries consist of time in bed (TIB), total sleep time (TST), sleep efficiency (SE), sleep latency (SL), number of awakenings (NAWK), wake after sleep onset (WASO).

### Data Analyses

Statistical analyses were performed using SPSS 13.0 and JASP version 0.14.1. Since our data did not meet the assumption for parametric tests, in particular the assumption of normally distributed data, and due to small sample size, we used non-parametric tests. Mann–Whitney U test were employed to evaluate differences between insomnia patients and healthy controls for all the questionnaires evaluating insomnia severity, chronotype, daytime sleepiness, sleep-related metacognitions, sleep-related dysfunctional beliefs, stress, global health, anxiety and depression symptoms. Due to the difference in age between patients and controls, this variable was used as covariate for all the comparisons in a rank analysis of covariance (Quade, 1967). To test differences before and after treatment, we used Wilcoxon signed-rank test with questionnaires assessing insomnia severity, sleep-related cognition and metacognitions, and sleep diaries indices as dependent variables. We also divided patients with insomnia into two groups based on their MCQ-I scores after treatment in order to identify those patients who still presented sleep-related metacognitive impairment (high MCQ-I=MCQ-I total score >110) and those without sleep-related metacognitive impairment (low MCQ-I=MCQ-I total score ≤110) after CBT-I, and to test possible differences between these two groups. Moreover, we extracted DBAS and MCQ-I delta scores (difference between pre- and post-treatment scores) to further investigate the effects of CBT-I on sleep-related cognition and metacognition in patients with high and low MCQ-I after treatment. Correlations between variables were assessed by means of Spearman’s correlation coefficient. Chi-Square Test was used to assess gender and chronotype differences between patients with insomnia and controls. Significance levels were set at *p*<0.05.

## Results

### Healthy Controls vs. Insomnia Patients

Comparisons between insomnia patients and healthy controls for demographic and clinical data are reported in Table 1.

**Table 1:** Comparison (Mann–Whitney U test or Chi Square test for gender and chronotype) between healthy control and insomnia patients in scores’ questionnaire.

Baseline indices (mean±SD)	Healty controls	Insomnia patients	*Z* or *χ*^2^	Sig.
Gender	*F* =63% M=37%	*F* =51.9% M=48.1%	0.68^*^	0.409
Age	33.00±13.77	46.37±13.67	−2.89	<0.001
ESS	6.44±3.23	4.41±3.17	−1.54	0.119
ISI	4.59±2.86	14.67±4.67	−5.88	<0.001
DBAS	51.52±21.07	91.41±17.25	−5.56	<0.001
Circadian typology (MEQ)	Morning-type: 3.7%	Morning-type: 14.8%		
	Intermediate-type: 74.1%	Intermediate-type: 66.7%		
	Evening-type: 22.2%	Evening-type: 18.5%	1.99^*^	0.368
BDI-I	8.37±7.22	11.04±5.20	−2.32	<0.05
PSS	17.19±5.23	18.00±4.72	−0.77	0.231
STAI Y-1	42.48±8.28	43.48±10.52	−0.29	0.605
STAI Y-2	42.37±7.61	44.19±8.59	−0.77	0.130
MCQ-I	105.63±20.52	138.11±26.23	−4.35	<0.001
GHQ	15.93±3.06	17.22±4.63	−0.93	0.171

SD, standard deviation; ESS, Epworth sleepiness scale; ISI, insomnia severity index; DBAS (16 item), dysfunctional beliefs and attitudes about sleep; MEQ-SA, morningness-eveningness questionnaire self-assessment; BDI-I, beck’s depression inventory for insomnia; PSS, perceived stress scale; STAI (Form Y-1 and Y-2), state– trait anxiety inventory; MCQ-I, metacognition insomnia questionnaire; and GHQ, general health questionnaire.

* *χ*^2^.

**Figure 1:**
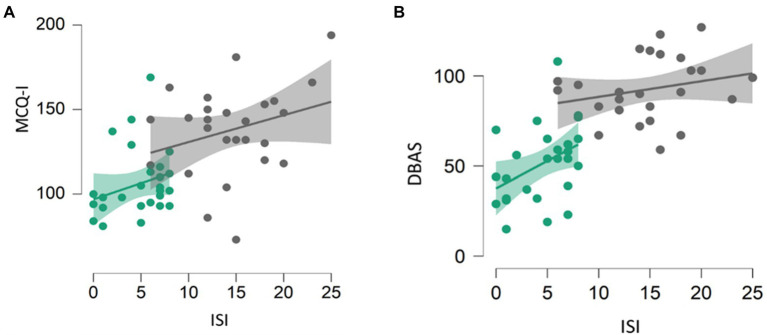
Correlation between MCQ-I and insomnia severity **(A)** and DBAS and insomnia severity **(B)**. Healthy subjects are represented by green dots, patients by black dots. DBAS (16 item), dysfunctional beliefs and attitudes about sleep; MCQ-I, metacognition insomnia questionnaire; and ISI, insomnia severity index.

A significant positive correlation was found between MCQ-I and ISI (spearman’s rho=0.577, *p*<0.001; Figure 1A), and between DBAS and ISI (spearman’s rho=0.717, *p*<0.001) scores in the whole sample (Figure 1B).

### Effect of CBT-I on Insomnia Symptoms, Sleep Diaries, and Clinical Indices

The effects of CBT-I on insomnia symptoms, sleep diaries and clinical indices are reported in Tables 2 and 3. As expected, we observed a significant improvement of insomnia severity both on ISI score and sleep diaries.

**Table 2:** Comparison (Wilcoxon signed rank test) between pre- and post-treatment questionnaire’s scores.

Questionnaire (mean±SD)	Pre-treatment	Post-treatment	*Z*	Sig.
ESS	4.41±3.17	4.74±3	−0.50	NS
ISI	14.67±4.67	7.48±4.29	−4.32	<0.001
DBAS	92.41±17.25	50.15±24.35	−4.36	<0.001
BDI	11.04±5.20	3.33±3.32	−4.37	<0.001
PSS	18±4.72	12.19±4.68	−3.98	<0.001
STAY-1	43.48±10.52	35.26±12.24	−2.99	0.003
STAY-2	44.19±8.59	34.82±9.07	−4.29	<0.001
MCQ-I	138.11±26.23	123.70±28.62	−2.65	0.008
GHQ	17.22±4.63	9.07±5.58	−4.08	<0.001

ESS, Epworth sleepiness scale; ISI, insomnia severity index; DBAS (16 item), dysfunctional beliefs and attitudes about sleep; MEQ-SA, morningness-eveningness questionnaire self-assessment; BDI-I, beck’s depression inventory for insomnia; PSS, perceived stress scale; STAI (Form Y-1 and Y-2), state– trait anxiety inventory; MCQ-I, metacognition insomnia questionnaire; and GHQ, general health questionnaire.

**Table 3:** Comparison (Wilcoxon signed rank test) between pre- and post-treatment sleep diaries’ data.

Sleep diaries data (mean±SD)	Pre-treatment	Post-treatment	*Z*	Sig.
SL (min)	35.39±36.94	16.76±12.24	−3.48	<0.001
N awk	1.85±1.31	1.09±1.18	−3.62	<0.001
WASO (min)	34.21±33.77	20.07±24.67	−2.40	0.011
TIB (min)	481.96±33.25	429.73±50.72	−4.19	<0.001
TIB (h)	8.03±0.55	7.16±0.85	−4.19	<0.001
TST (min)	368.42±77.94	382.55±56.82	−1.21	N.S.
TST (h)	6.14±1.3	6.38±0.95	−1.23	N.S.
SE (%)	76.44±14.61	88.88±7.27	−3.79	<0.001

SL, sleep Latency; N awk, number of awakenings; WASO, wake after sleep onset; TIB, time in bed; TST, total sleep time; SE, sleep efficiency; and N.S., not significant.

### Effect of CBT-I on Metacognition

**Figure 2:**
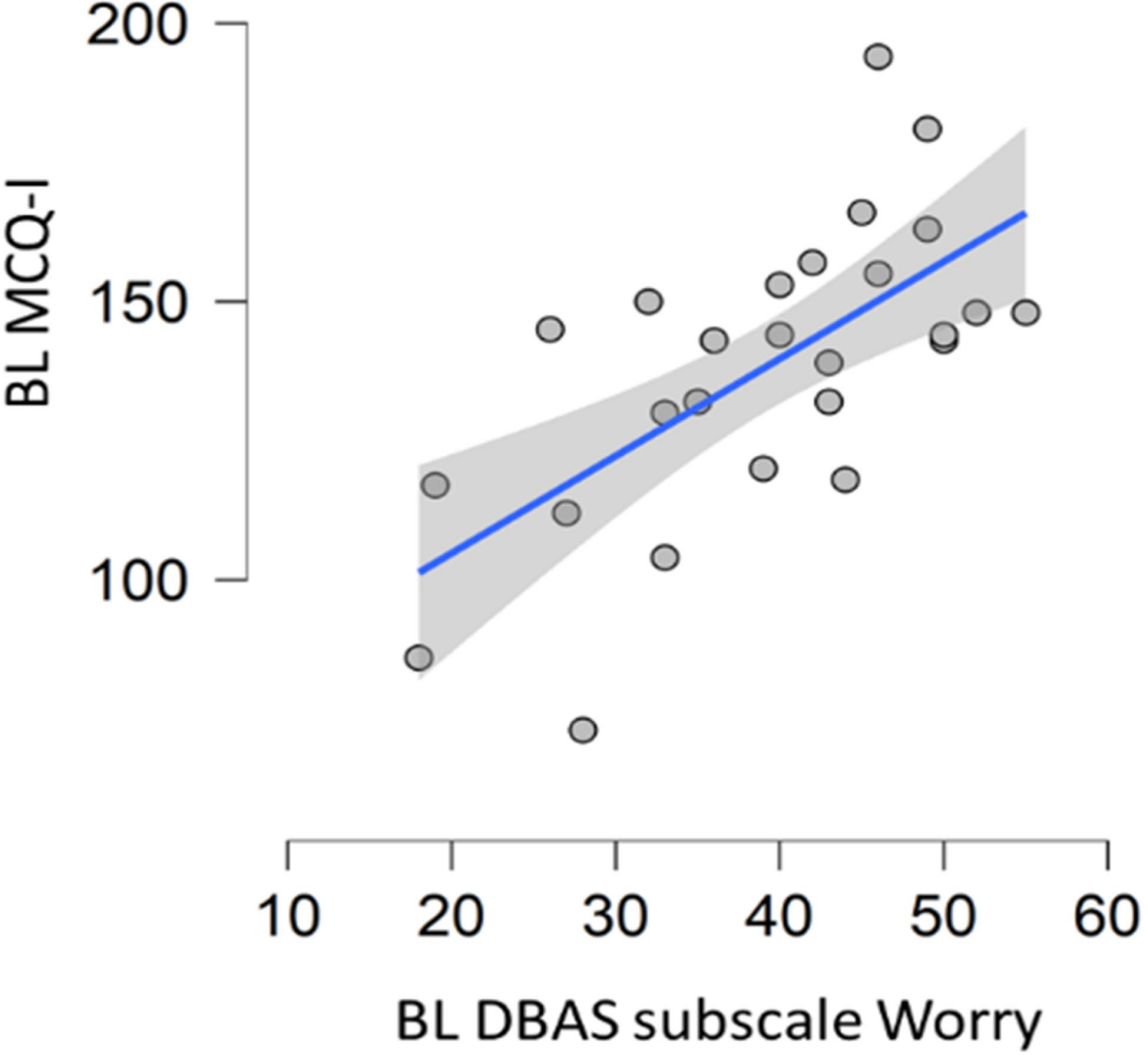
Correlation between baseline metacognition scores and DBAS subscale assessing worrying. DBAS (16 item), dysfunctional beliefs and attitudes about sleep; MCQ-I, metacognition insomnia questionnaire; and BL, baseline.

Significant correlation was found between MCQI total score and DBAS subscale “worry/helplessness about sleep” (spearman’s rho=0.625, *p*<0.001; Figure 2). No correlation between MCQI total score and DBAS total score was observed.

In spite of a significant effect of CBT-I in reducing both DBAS (DBAS_before CBT-I_=91.73±17.21 vs. DBAS_after CBT-I_=52.09±22.64; Sig=*p*<0.001) and MCQI (MCQ-I_before CBT-I_=138.11±26.23 vs. MCQ-I_after CBT-I_=123.70±28.62; Sig=*p*<0.05) total scores, 29.6% of patients maintained equal or worse MCQ-I score after treatment in comparison to baseline. Moreover, 63% of patients (17 out of 27) showed MCQ-I scores above the cutoff following CBT-I intervention.

**Figure 3:**
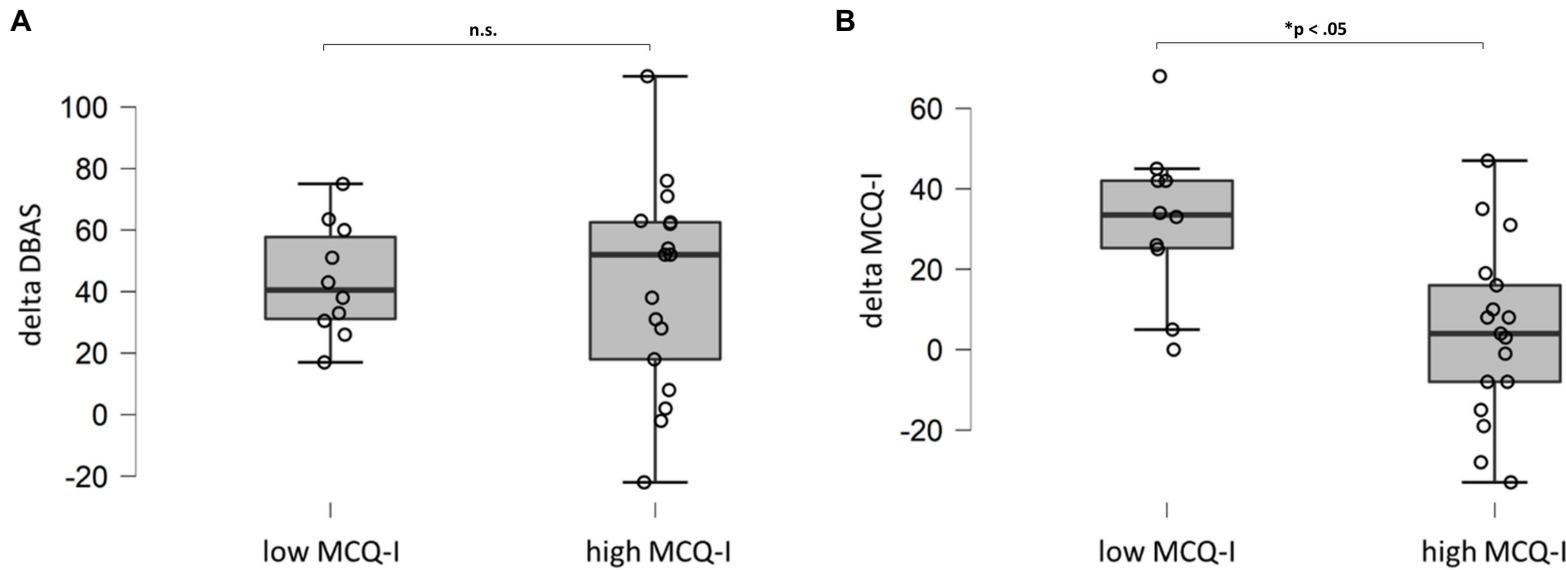
Comparison (Mann–Whitney U test) for DBAS **(A)** and MCQ-I **(B)** delta scores for patients with MCQ-I score above or below the cut-off after treatment. DBAS (16 item), dysfunctional beliefs and attitudes about sleep; MCQ-I, metacognition insomnia questionnaire; and n.s., not significant.

Sub-dividing the insomnia sample in patients who still presented significant levels of metacognitive deficits after CBT-I and who did not, and evaluating delta score (difference between pre- and post-treatment scores) of DBAS and MCQI, we found a significant difference in MCQ-I delta scores. In other words, patients with severe metacognitive impairment at end of the treatment did not receive a beneficial effect of CBT-I on this dimension (Figure 3B; high MCQ-I). On the contrary, DBAS showed important and significant reduction in both groups (Figure 3A).

**Figure 4:**
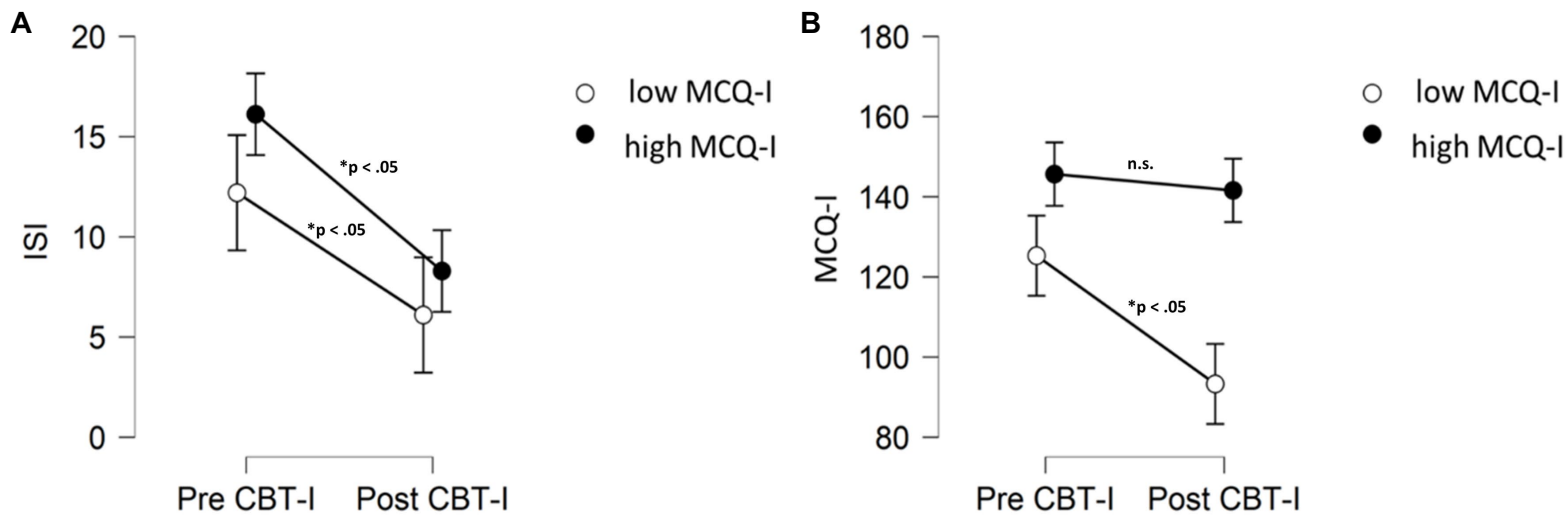
Comparisons (Wilcoxon signed rank test) in ISI **(A)** and MCQ-I **(B)** scores before and after treatment for patients MCQ-I score above or below the cut-off after CBT-I. MCQ-I, metacognition insomnia questionnaire; ISI, insomnia severity index; and n.s., not significant.

Interestingly, although patients who still presented metacognitive impairment received significant beneficial effects of CBT-I on both insomnia symptoms and dysfunctional beliefs, they did not improve their MCQ-I after treatment in comparison to baseline (*p*>0.05; Figure 4).

## Discussion

The aim of this study was to confirm the presence of impairment in sleep-related metacognition and to evaluate the effect of CBT-I on metacognitive functioning in patients with insomnia disorder. As expected, we confirmed the presence of dysfunctional metacognition in patients with insomnia in comparison to healthy controls. Regarding CBT-I, we found that metacognitive dysfunctions received beneficial effect from the treatment as measured by MCQ-I. These improvements were associated with a significant reduction in insomnia symptoms quantified both by sleep diaries’ variables and by ISI scores. However, results concerning metacognition were not homogeneous. Indeed, we found a significant percentage of patients (63%) who did not present MCQ-I scores below the cut-off after CBT-I, in spite of a significant amelioration of insomnia symptoms. In other words, it seems that for some patients CBT-I is not sufficient for treating their maladaptive metacognitive functioning, even if the treatment is successful, as underlined by ISI scores (Figure 4). Importantly, CBT-I is strongly effective in reducing dysfunctional beliefs and thoughts regarding sleep, as measured by DBAS, also in patients who still presented significant levels of impaired metacognition (Figure 3).

Metacognition is the ability to reflect on one’s own mental states and cognitive processes, allowing subjects to regulate mental state, beliefs and processes (Flavell, 1976). A crucial aspect of metacognition in cognitive psychotherapy is to shift the focus from thought contents to cognitive processes (e.g., repetiveness and incontrollability). In the context of insomnia, a metacognitive model has been proposed by Ong et al. (2012). According to this model, the authors conceptualized two different levels of cognitive arousal that reflect a distinction between cognition and metacognition. “Primary arousal” refers to cognitive contents and beliefs regarding sleep that directly interfere with sleep. “Secondary arousal” instead consists of the cognitive process in response to thoughts and cognition, such as worry and rumination focused on beliefs that amplifies primary arousal. The dysfunctional response to cognition, represented for example by rumination and by implicit beliefs regarding its usefulness, clearly interferes with the automatic regulation of sleep throughout the generation and maintenance of hyperarousal. In our study, we considered DBAS scores, a scale developed to evaluate sleep-disruptive cognitions (Morin et al., 2007), as representative of “primary arousal,” whereas MCQ-I referred to “secondary arousal.” Confirming previous literature (Palagini et al., 2014), we found that patients showed significantly higher levels of MCQ-I and DBAS in comparison to controls. Importantly, these scales had significant strong correlations with insomnia symptoms when considering the whole sample, confirming the impact of these maladaptive cognitive beliefs and processes on the severity of the disorder.

Interestingly, when considering only the group of insomnia patients, the total scores of these two scales were not correlated. The only significant correlation was found between MCQ-I and DBAS subscale related to worry, confirming the latter as part of metacognitive functioning. Our results concerning CBT-I intervention suggest a dissociation between cognition and metacognition in treatment response. Indeed, despite we found significant global improvement in both DBAS and MCQ-I, more than a half of patients (63%) showed MCQ-I scores above the cut-off after intervention. Specific comparison analyses between patients with and without metacognitive impairment revealed that patients with MCQ-I scores above the cut-off after CBT-I received a significant improvement on insomnia symptoms but not on sleep-related metacognitive dysfunction, in comparison to patients with MCQ-I below the cut-off. Importantly, by further analysing delta DBAS and delta MCQ-I scores (difference between pre- and post-treatment scores), we found that the two groups specifically differ in terms of metacognition in response to treatment. Indeed, delta scores did not differ for DBAS but showed significant difference for MCQ-I (Figure 3). This finding might be interpreted on the basis of the specific cognitive intervention embedded in CBT-I package. Indeed, cognitive intervention associated to this therapy is thought to challenge dysfunctional sleep-related beliefs in order to decrease anxiety and arousal caused by these thoughts (Perlis et al., 2005). In other words, CBT-I might act specifically on “primary arousal” but not “secondary arousal,” i.e., metacognition. However, we have to consider that also patients with significant metacognitive impairment after treatment (those patients who still present MCQ-I score above the cut-off of 110) showed a significant reduction of insomnia symptoms, hence it seems that this residual “secondary arousal” is not able to hinder the effect of CBT-I. One possible explanation in discussing this result, is that the behavioural core component of the intervention, such as sleep restriction, considered one of the most active element of CBT-I (Miller et al., 2014), might drive this improvement. Accordingly, a recent randomized controlled trial underlined the centrality of sleep restriction in improving insomnia symptoms throughout sleep consolidation (Maurer et al., 2020). Nevertheless, the presence of patients who still presented dysfunctional metacognition after treatment should be carefully considered. Indeed, this might result crucial to identify those patients at risk for relapses in the long term, thus providing fundamental prognostic information for clinicians. The presence of metacognitive dysfunction in insomnia patients after CBT-I may favor the re-emergence of uncontrollable maladaptive strategies, such as worry and rumination, thus leading to relapses. If this holds true, adding a specific component, addressing dysfunctional metacognition, might prevent relapses in those patients (Hjemdal et al., 2019).

Several works indicate that dysfunctional metacognitive functioning and related coping strategies are typical features of insomnia disorder. Patients with insomnia frequently employ thought control strategies, such as reappraisal, worry and thought suppression, at bedtime (Harvey, 2001; Ellis and Cropley, 2002). Furthermore, they also have more positive beliefs about worry. For example statements like “I will lose out in life if I do not use the time before falling asleep to think through things,” “Helps me get things sorted out in my mind,” “I need to use the time in bed before sleeping to think in order to remain organised,” or “Helps me solve problems,” regarding pre-sleep worry are more frequently endorsed by patients with insomnia in comparison to good sleepers (Harvey, 2003). These metacognitive beliefs and coping strategies are known to boost further thought intrusions (Wegner, 1994) maintaining sleep disturbances. Several techniques, such as attention training techniques, detached mindfulness and metacognitive-focused Socratic dialogue, are effective and well established in the context of metacognitive therapy (Wells, 2009a). The addition of these interventions in CBT-I protocol should be carefully evaluated in order to target a specific psychopathological functioning that is a hallmark of patients affected insomnia.

When interpreting the findings of our study several limitations should be considered. First, the small sample size and the lack of a control group does not allow us for a robust conclusion and generalization of our results. Secondly, the lack of a follow-up limits the possibility to evaluate the maintenance of improvements in insomnia symptoms and or amelioration of metacognitive functioning in the long term.

In conclusion, our study showed that patients, who still presented metacognitive impairment after treatment, received significant beneficial effects by CBT-I on both insomnia symptoms and dysfunctional beliefs, but not on metacognition, suggesting that this dimension should be carefully evaluated in insomnia patients, since it might have a prognostic valence. Indeed, although high levels of dysfunctional sleep-related metacognition do not seem to hinder the beneficial effect of CBT-I, they might represent a risk factor for relapses in the long term. Future studies should adopt a long term follow-up in order to test this hypothesis. Furthermore, the insertion of specific interventions focused on metacognition, such as attentional training, detached mindfulness as well as metacognitive-focused Socratic dialogue (Wells, 2009b), might be considered.

## Data Availability Statement

The datasets presented in this article are not readily available because the participants did not provide informed consent for sharing data. Requests to access the datasets should be directed to andrea.galbiati.unisr@gmail.com.

## Ethics Statement

The studies involving human participants were reviewed and approved by San Raffaele Hospital (Milan, Italy) Ethics Committee. The patients/participants provided their written informed consent to participate in this study.

## Author Contributions

AG: study design, management, and manuscript writing and analysis. MS: study design, data collection, and manuscript drafting and analysis. ASc, ASa, CL, GD’E, and SM: study design, data collection, and manuscript drafting. LF-S: study design and management. VC: study design, management, and manuscript drafting. All authors contributed to the article and approved the submitted version.

## Conflict of Interest

The authors declare that the research was conducted in the absence of any commercial or financial relationships that could be construed as a potential conflict of interest.

## Publisher’s Note

All claims expressed in this article are solely those of the authors and do not necessarily represent those of their affiliated organizations, or those of the publisher, the editors and the reviewers. Any product that may be evaluated in this article, or claim that may be made by its manufacturer, is not guaranteed or endorsed by the publisher.
